# Altered circulating CCR6^+^and CXCR3^+^ T cell subsets are associated with poor renal prognosis in MPO-ANCA-associated vasculitis

**DOI:** 10.1186/s13075-021-02576-x

**Published:** 2021-07-21

**Authors:** Zhonghua Liao, Jiale Tang, Liying Luo, Shuanglinzi Deng, Lisa Luo, Fangyuan Wang, Xiangning Yuan, Xinyue Hu, Juntao Feng, Xiaozhao Li

**Affiliations:** 1 Department of Nephrology, Xiangya Hospital, Central South University, Changsha, 410008 Hunan China; 2 Department of Respiratory and Critical Care Medicine, Key Cite of National Clinical Research Center for Respiratory Disease, Xiangya Hospital, Central South University, Changsha, 410008 Hunan China

**Keywords:** Antineutrophil cytoplasmic antibody (ANCA)-associated vasculitis, Myeloperoxidase, Effector memory T cells, Kidney survival

## Abstract

**Background:**

Effector memory T cells are pivotal effectors of adaptive immunity with enhanced migration characteristics and are involved in the pathogenesis of ANCA-associated vasculitis (AAV). The diversity of effector memory T cells in chemokine receptor expression has been well studied in proteinase 3 (PR3)-AAV. However, few studies have been conducted in myeloperoxidase (MPO)-AAV. Here, we characterized chemokine receptor expression on effector memory T cells from patients with active MPO-AAV.

**Methods:**

Clinical data from newly diagnosed MPO-AAV patients and healthy subjects were collected and analyzed. Human peripheral blood mononuclear cells (PBMCs) isolated from patients with active MPO-AAV were analyzed by flow cytometry. The production of effector memory T cell-related chemokines in serum was assessed by ELISA.

**Results:**

We observed decreased percentages of CD4^+^ and CD8^+^ T cells in the peripheral blood, accompanied by a significant decrease in CCR6-expressing T cells but an increase in CXCR3^+^ T cells, in active MPO-AAV. Furthermore, the decrease in CCR6 and increase in CXCR3 expression were mainly limited to effector memory T cells. Consistent with this finding, the serum level of CCL20 was increased. In addition, a decreasing trend in the T_EM_17 cell frequency, with concomitant increases in the frequencies of CD4^+^ T_EM_1 and CD4^+^ T_EM_17.1 cells, was observed when T cell functional subsets were defined by chemokine receptor expression. Moreover, the proportions of peripheral CD8^+^ T cells and CD4^+^ T_EM_ subsets were correlated with renal prognosis and inflammatory markers.

**Conclusions:**

Our data indicate that dysregulated chemokine receptor expression on CD4^+^ and CD8^+^ effector memory T cells and aberrant distribution of functional CD4^+^ T cell subsets in patients with active MPO-AAV have critical roles related to kidney survival.

## Background

Antineutrophil cytoplasmic antibody (ANCA)-associated vasculitis (AAV) is classified as proteinase 3 (PR3)-AAV and myeloperoxidase (MPO)-AAV according to the difference in ANCA serotype. MPO-AAV has been reported to be more common than PR3-AAV in southern Europe, Asia, and the Pacific, except for New Zealand and Australia [1]. The disease spectrum of Chinese AAV patients and the target antigens of ANCAs are quite different from those in Western countries, and MPO-AAV is the dominant form of AAV in China [2, 3].

Accumulating evidence has demonstrated that T cells are involved in the pathogenesis of AA V[4]. There was an abundance of infiltrating T cells in renal biopsies from AAV patients. In the kidney, the number of CD8^+^ T cells was approximately the same as the number of CD4^+^ T cells, and a higher number of CD8^+^ T cells in the renal interstitial infiltrate was correlated with a lower eGFR [5]. Additionally, anti-T cell treatment and anti-thymocyte globulin have shown beneficial therapeutic effects in patients with severe AAV [6, 7]. Moreover, in experimental anti-MPO-associated crescentic glomerulonephritis, mice depleted of CD4^+^ T cells and CD8^+^ T cells developed significantly improved renal prognosis [8, 9]. In accordance with these findings, targeting cytokines associated with the differentiation of CD4^+^ T helper cell subsets was effective in improving anti-MPO glomerulonephritis [10]. Therefore, T cells play an important role in AAV.

Based on the distinctive pattern of activation and effector functions, human T cells include naïve T cells (T_naïve_), effector T cells, and central memory T (T_CM_) cells and effector memory T (T_EM_) cells [11]. Naive lymphocytes are functionally quiescent; once they encounter foreign antigens, they proliferate and differentiate into effector T lymphocytes that eliminate the pathogen [12]. However, the majority of differentiated effector T lymphocytes are short-lived and are eliminated later; a small fraction survives as memory T cells, which are reactivated quickly and differentiate into T_EM_ cells upon a subsequent encounter with the antigen, leading to efficacious secondary responses [13]. T_EM_ cells play the main role in the immune response, with enhanced functional activation and migration properties [14]. Chemokine receptors play an important role in mediating T cell recruitment to the site of inflammations. The expression of CCR4, CCR6, and CXCR3 on memory T cells reflects their diverse capacities for trafficking to non-lymphoid tissues, including the kidney, spleen, lung, liver, and gut [15, 16]. Fagin et al. reported increases in the proportions of CCR4^+^ and CCR6^+^ CD4^+^ T cells in the peripheral blood of patients with PR3-AAV, and the increases in CCR4 and CCR6 expression were largely limited to T_CM_ and T_EM_ cells [17]. However, the expression of CCR4, CCR6, and CXCR3 on CD4^+^ and CD8^+^ T cells in MPO-AAV patients is relatively uncharacterized.

In PR3-AAV, several studies have demonstrated that CD4^+^ T_EM_ cells are involved in autoimmune pathologies [18–20]. Persistent expansion of CD4^+^ T_EM_ cells was observed during remission, while CD4^+^ T_EM_ cells were decreased during the active phase because of migration to the site of inflammation in PR3-AAV [18, 20]. In addition, further research on the distribution of circulating CD4^+^ T_EM_ cell subsets during PR3-AAV remission identified that CD4^+^ T_EM_17 cells were positively associated with the number of organs involved, whereas CD4^+^ T_EM_1 cells were negatively associated with the number of organs involved [19]. However, CD4^+^ T_EM_ cells have not been well studied in MPO-AAV patients, and little data are available regarding the distribution of CD8^+^ T_EM_ cells in AAV.

Our study was designed to clarify the distribution of circulating CD4^+^ T_EM_ cells and CD8^+^ T_EM_ cell subsets based on chemokine receptor expression in MPO-AAV patients.

## Methods

### Study population

Complete data for 143 patients with active MPO-AAV (69 women, 74 men; age range: 52 to 86 years) and 176 healthy control (HC) (90 women, 86 men; age range: 40 to 75 years) were collected during health examinations at Xiangya Hospital between December 2012 and June 2020. The clinical characteristics of the MPO-AAV patients and healthy subjects are reported in Table 1. For subsequent experiments, the peripheral blood was collected from 33 patients with active MPO-AAV and 20 HC at Xiangya Hospital. Clinical data are reported in Table 2.

**Table 1:** Laboratory and clinical characteristics of newly diagnosed active MPO-AAV patients and healthy control before treatment

	Active MPO-AAV	HC
n	143	176
Age (Y), mean (range)	60 (52–86)	53 (40–75)
Gender (F/M)	69/74	90/86
MPO-ANCA titer, (median, range)	94.97 (18.37–167.98)	-
White blood cells (10^9^/l) (mean, SD)	8.70±3.57	6.05±1.11
Neutrophil (10^9^/l) (mean, SD)	6.81±3.36	3.49±0.83
Neutrophil percentage (%) (mean, SD)	76.22±9.46	57.14±7.40
Lymphocyte (10^9^/l) (mean, SD)	1.10±0.54	2.01±0.45
Lymphocyte percentage (%) (mean, SD)	14.09±7.39	33.46±5.74

**Table 2:** Patients’ clinical and biological features at time of blood sampling

	Active MPO-AAV	HC
n	33	20
Age (Y), mean (range)	63 (50–82)	59 (33–79)
Gender (F/M)	16/17	11/9
MPO-ANCA titer (median, range)	87.44 (28.48–164.5)	
BVAS (mean, range)	19(15–27)	
White blood cells (10^9^/l) (mean, SD)	7.52±3.05	
Hemoglobin (g/l) (mean, SD)	71.82±16.84	
Platelet (10^9^/l) (mean, SD)	240.58±68.26	
Neutrophil (10^9^/l) (mean, SD)	5.65±2.87	
Neutrophil percentage (%) (mean, SD)	73.67±10.43	
Lymphocyte (10^9^/l) (mean, SD)	1.08±0.65	
Lymphocyte percentage (%) (mean, SD)	15.71±8.88	
CRP (mg/L) (median range)	37.2 (1.45–253)	
ESR (mm/h) (median range)	95 (2–120)	
Clinical manifestation**,** n (%)		
ENT	2 (6%)	
Eyes	2 (6%)	
Pulmonary	31 (94%)	
Gastrointestinal	17 (52%)	
Nervous system	1 (3%)	
Renal	33 (100%)	
Proteinuria, n (%)	31 (94%)	
Proteinuria(g/day) (median range)	1.6 (0.48–6.87)	
Heterogeneous hematuria, n (%)	30 (97%)	
Renal insufficiency, n (%)	33 (100%)	
Serum creatine(umol/L) (median range)	628 (124–1395)	
eGFR (ml/min*1.73 m2) (median range)	6.3 (2.3–58)	
Renal pathological data		
Subjects, no.	14	
Classification, n%		
Focal	1 (7.1%)	
Mixed	2 (14.3%)	
Crescentic	8 (57.1%)	
Sclerotic	3 (21.4%)	

All patients with MPO-AAV included in our study were newly diagnosed according to the definition established at the Chapel Hill Conference [21]. Clinical data were collected prior to the use of glucocorticoids, immunosuppressants, and plasma exchange. In addition, the Birmingham Vasculitis Activity Score (BAVS) of all patients before the time of sample collection was greater than 15, suggesting that vasculitis was in the active stage [22]. Patients were excluded if they were administered plasma exchange, glucocorticoid treatment, or immunosuppressive treatment or had infection, neoplasms, or concomitant immune system diseases. ESRD was defined by dialysis dependence for more than 3 months.

### Flow cytometry

Venous blood samples were collected in EDTA tubes from patients with active MPO-AAV and HC. To obtain the best detection results and minimize cell manipulation, our peripheral blood samples were processed within 4 h and immediately analyzed by FACS. Peripheral blood mononuclear cells (PBMCs) were isolated from the peripheral blood on Ficoll Paque-PLUS (GE Healthcare, Little Chalfont, UK) by density gradient centrifugation. Freshly collected PBMCs were stained immediately using the following fluorochrome-conjugated anti-human antibodies: BV510-conjugated anti-CD3, BB515-conjugated anti-CD4, APC-Cyanine7 (APC-Cy7)-conjugated anti-CD8, PerCP/Cyanine5.5-conjugated anti-CD45RO, PE/Cyanine7 (PE-Cy7)-conjugated anti-CCR7, PE-conjugated anti-CCR6, Brilliant Violet 421-conjugated anti-CCR4, and APC-conjugated anti-CXCR3 (BD Biosciences). Briefly, appropriate concentrations of fluorochrome-conjugated monoclonal antibodies specific for cell surface antigens were added to tubes containing 100 μL of PBMCs and incubated for 30 min in the dark at 4°C. Subsequently, cells were washed twice with phosphate-buffered saline/0.01% bovine serum albumin and immediately analyzed using a BD FACS Canto II flow cytometer.

### Enzyme-linked immunosorbent assay

Serum CCL20 (Boster Biological Technology) levels were determined by enzyme-linked immunosorbent assay (ELISA) according to the manufacturer’s protocols.

### Statistical analysis

Data are expressed as the mean ± SEM values. To compare differences between two groups when the continuous data fit a normal distribution, an unpaired Student’s t test was used. To compare differences in nonparametric data between groups, the Mann–Whitney U test was used. For correlation analysis, the Spearman correlation coefficient was used to examine variables that did not obey a normal distribution. A *P* value less than 0.05 was considered to indicate a significant difference.

## Results

### Result 1: CD4^+^ T cells and CD8^+^ T cells were significantly decreased in the peripheral blood of patients with active MPO-AAV

**Fig. 1:**
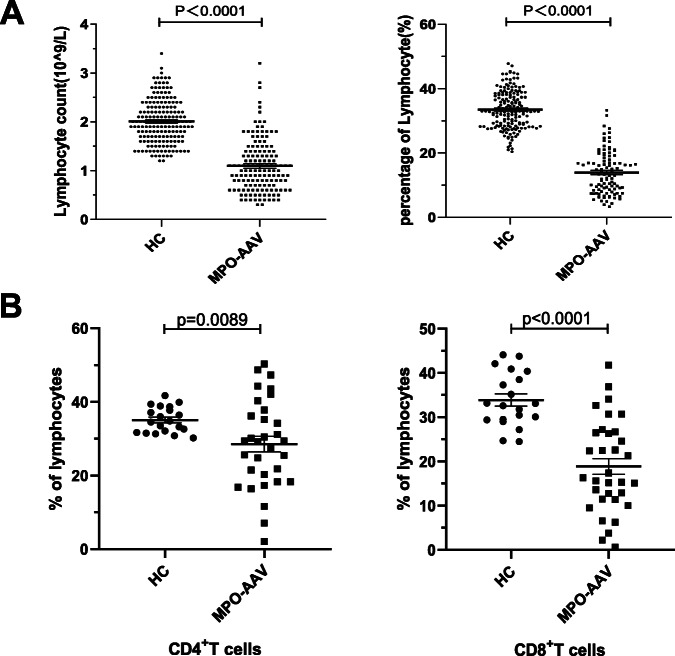
The distribution of lymphocyte between HC and active MPO-AAV patients. **A** Significant differences in the lymphocytes in the blood of active MPO-AAV patients and HC were observed. **B** According to the expression differences of T lymphocyte surface markers, CD4^+^ and CD8^+^T cell were further studied. Decreased frequencies of CD4^+^ and CD8^+^ T cells were detected in active MPO-AAV patients (n MPO-AAV=33 n HC =20)

Lymphopenia has been reported in the active stage of PR3-AAV, but whether lymphopenia also exists in patients with MPO-AAV remains to be studied [23]. Here, we analyzed the number and percentage of lymphocytes in routine blood tests of patients with active MPO-AAV and HC. As shown, significant decreases in the number and proportion of lymphocytes were observed in patients with active MPO-AAV compared to HC (Fig. 1A). T cells are the main type of lymphocytes, and both CD4^+^ T cells and CD8^+^ T cells have been noted to be involved in kidney injury [5]. To further identify the distribution of T cells, we evaluated the expression of CD3, CD4, and CD8 on the T cells and found that the proportions of CD3^+^CD4^+^CD8^−^ and CD3^+^CD4^−^CD8^+^ T lymphocytes were obviously reduced in the blood of active patients compared to HC (Fig. 1B). As in PR3-AAV, we confirmed the existence of lymphopenia, especially CD4^+^ T cells in MPO-AAV. Although the pathological effect of CD8^+^ T cells on the kidney has been demonstrated [5, 8], a decrease in CD8^+^ T cells in the peripheral blood of active MPO-AAV has rarely been reported.

### Result 2: Decreased frequency of CCR6^+^ T cells and increased frequency of CXCR3^+^ T cells in the peripheral blood of patients with active MPO-AAV

**Fig. 2:**
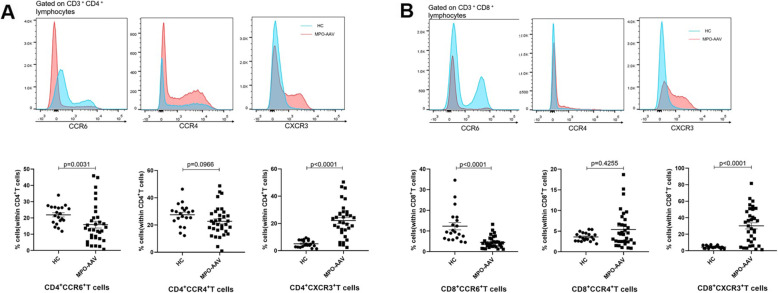
Decreased CCR6^+^ T cells and increased CXCR3^+^ T cells in active MPO-AAV patients. **A** PBMCs isolated from HC and active MPO-AAV patients were stained with PE-conjugated anti-CCR6, BV-421-conjugated anti-CCR4, and APC-conjugated anti-CXCR3 antibodies and then analyzed by flow cytometry (n MPO-AAV=33, n HC=20). The CD3^+^CD4^+^T cells were analyzed for surface expression of CCR4, CCR6, and CXCR3. Variation in percentages of CCR4^+^ and CXCR3^+^CD4^+^ T cells was observed between HC and active MPO-AAV patients. **B** Surface expression of CCR4, CCR6, and CXCR3 was also analyzed in CD3^+^CD8^+^ T cells. The percentages of CCR6^+^cells were decreased, and CXCR3^+^ cells were increased within the CD8^+^ T cell population in the peripheral blood of active MPO-AAV patients, when compared with HC

We hypothesized that the significant decrease in peripheral blood T lymphocytes in patients with active MPO-AAV may be related to the recruitment of activated T cells to sites of inflammation. Recruitment of T cells is closely related to the expression of chemokine receptors [14]. CCR4, CCR6, and CXCR3 are considered critical chemokine receptors involved in the recruitment of CD4^+^ and CD8^+^ T cells to sites of inflammation [24]. Thus, we analyzed the expression of CCR4, CCR6, and CXCR3 on CD4^+^ and CD8^+^ T cells. In contrast to Fagin’s findings in PR3-AAV [17], decreases in the percentages of CCR6-expressing cells within the CD4^+^ and CD8^+^ T cell populations were observed in patients with active MPO-AAV compared to HC, while no significant difference in CCR4-expressing T cells was found. In addition, an increased percentage of CXCR3-expressing T cells was first observed in our study. These differences may be attributed to disease activity and infiltration of inflammatory cells in the involved tissue in different stages of MPO-AAV (Fig. 2). Collectively, our data suggested that aberrant chemokine receptor expression may be related to the inflammatory trafficking of CD4^+^ and CD8^+^ T cells.

### Result 3: Lower frequencies of CCR6-expressing CD4^+^ T and CD8^+^ T memory cells and higher frequencies of CXCR3-expressing CD4^+^ memory T cells and CD8^+^ memory T cells were observed in the peripheral blood of MPO-AAV patients during the active stage

**Fig. 3:**
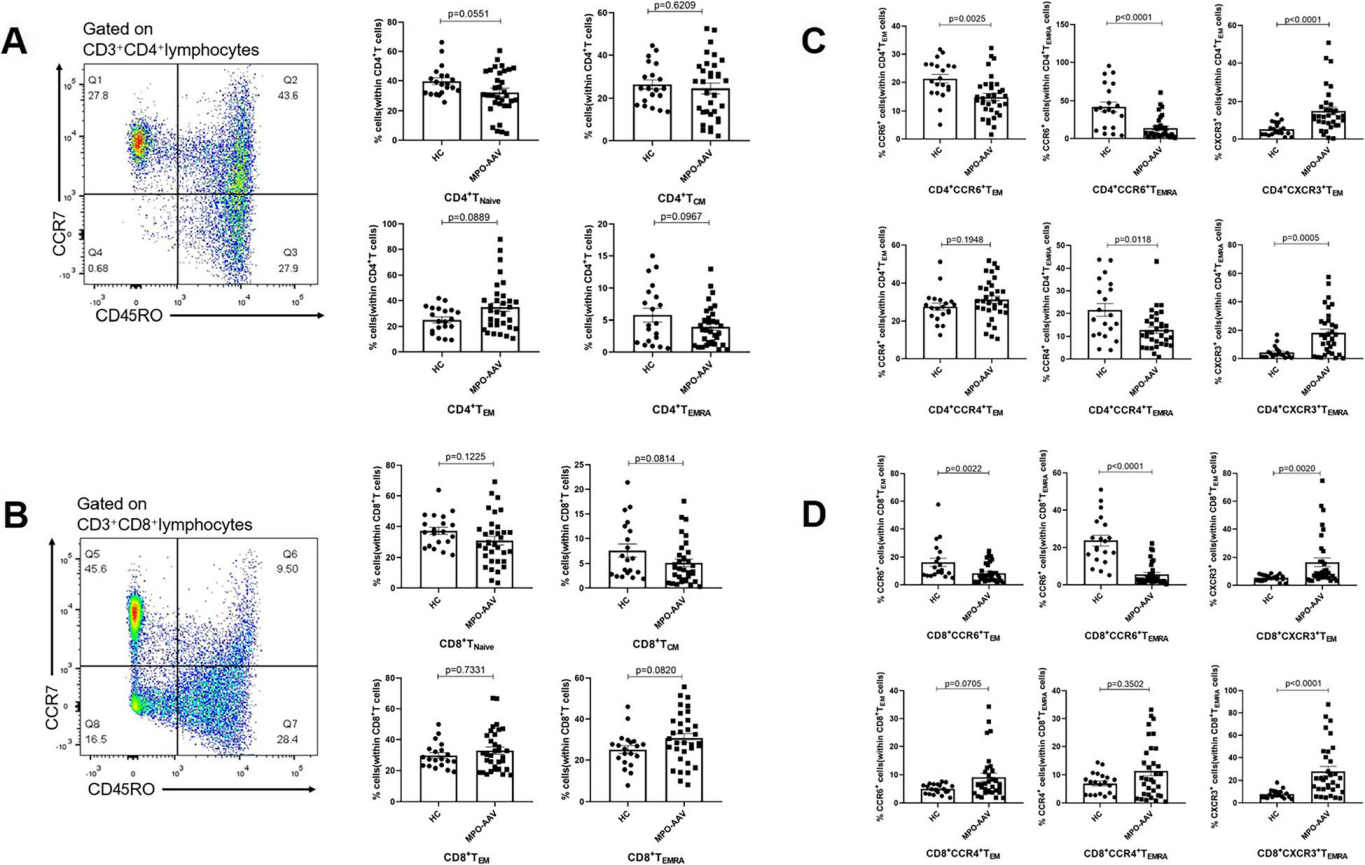
Surface expression of CCR4, CCR6, and CXCR3 on human T cell functional subset. **A**, **B** Representative plots and statistical data of CD4^+^T cell and CD8^+^T cell subsets. CD4^+^T cell subsets and CD8^+^ T cells subsets from the peripheral blood were identified according to the expression of CCR7 and CD45RO. The percentage of T_naïve_ (CCR7^+^CD45RO^−^), T_CM_ (CCR7^+^CD45RO^+^), T_EM_ (CCR7^-^CD45RO^+^), and T_EMRA_ (CCR7^−^CD45RO^−^) in the peripheral blood of HC and active MPO-AAV patients is shown (n MPO-AAV=33, HC=20). **C**, **D** Percentages of CCR6^+^ T cells, CCR4^+^ T cells CXCR3^+^ T cells in the T_EM_ (CCR7^−^CD45RO^+^), and T_EMRA_ (CCR7^−^CD45RO^−^) subpopulation were shown

Both the CD4^+^ T and CD8^+^ T cell populations consist of naïve lymphocytes and memory lymphocytes [11]. Effector memory T cells are cytotoxic and mediate protective immunity to bacterial and viral pathogens [25–27]. In many autoimmune diseases, the involvement of effector memory T cells in the local immune response via chemotactic pathways has been proven [18, 28–30]. Having found significant decreases in the frequencies of CCR6-expressing T cells and significant increases in CXCR3-expressing T cells in patients with MPO-AAV, we further clarified the phenotypic features of effector memory T cells based on chemokine receptor expression. Here, we analyzed the distribution of CD4^+^ and CD8^+^ functional subsets and the expression of CCR4, CCR6, and CXCR3 on CD4^+^ and CD8^+^ effector memory T cells. No significant differences were found in the CD4^+^ T cell and CD8^+^ T cell functional subpopulations between MPO-AAV patients and HC (Fig. 3A, B). In contrast with the finding in PR3-AAV [17], we observed significant declines in the CD4^+^CCR6^+^ T_EM_ and CD4^+^CCR6^+^ T_EMRA_ (effector memory T cells re-expressing CD45RA) populations in MPO-AAV. No significant difference was found in the CD4^+^CCR4^+^ T_EM_ population. In addition, significant increases were found in the CD4^+^CXCR3^+^ T_EM_ and CD4^+^CXCR3^+^ T_EMRA_ subsets. Consistent with the finding for CD4^+^ effector memory T cells, the expression of chemokine receptors on CD8^+^ T_EM_ and CD8^+^ T_EMRA_ cells showed the same trends (Fig. 3C, D). Based on these results, we speculated that the unbalanced expression of chemokine receptors on effector memory T cells probably indicates T cell recruitment into inflammatory tissues and the diversity in the T cell distribution during the early and late autoimmune responses in patients with MPO-AAV.

### Result 4: Increased frequencies of CD4^+^ T_EM_1 and CD4^+^ T_EM_17.1 cells in the peripheral blood of patients with active MPO-AAV

**Fig. 4:**
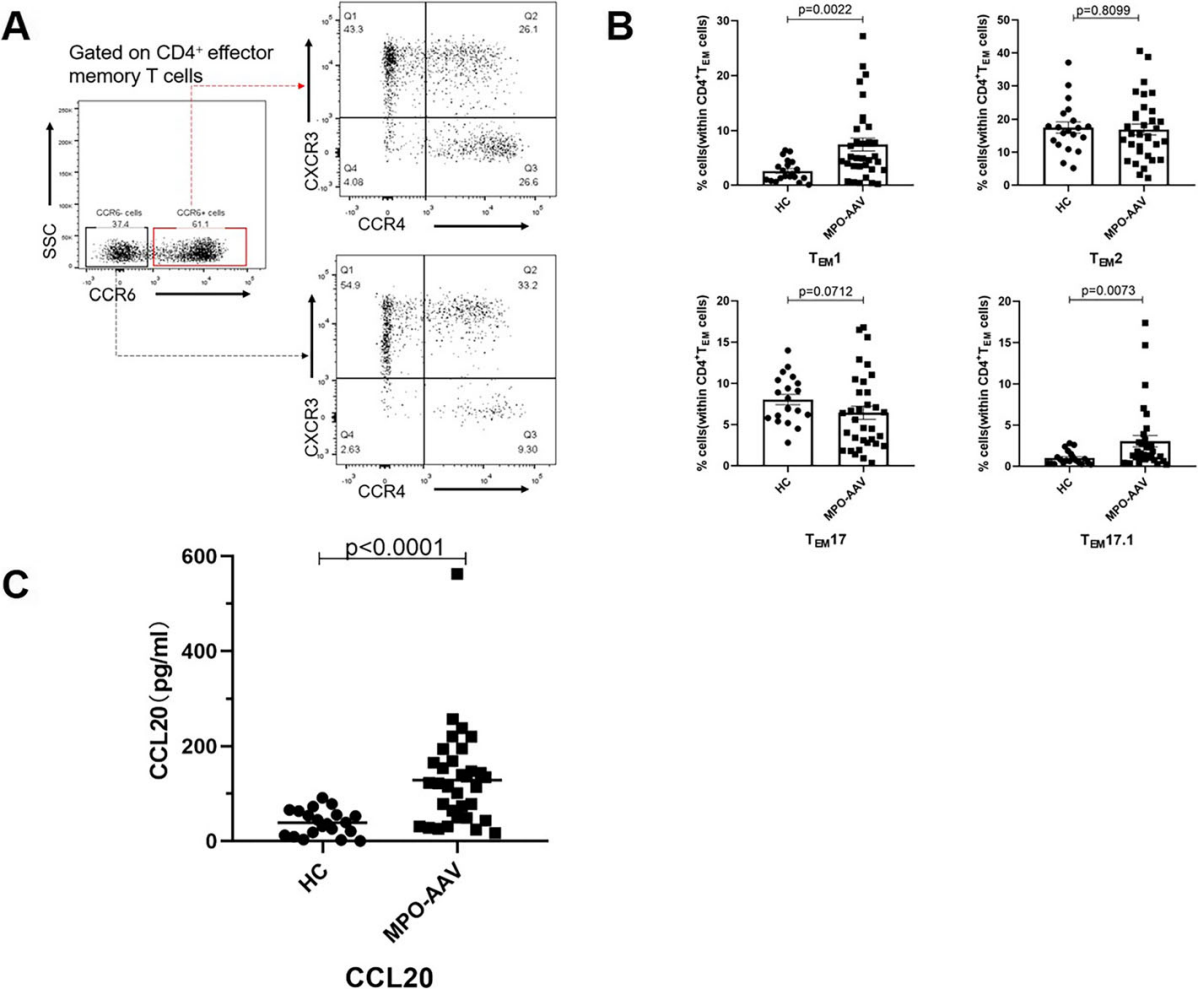
The distribution of CD4^+^ effector memory T cell subsets in HC and active MPO-AAV patients. **A** Representative plots of CD4^+^ effector memory T cell subsets. Gating strategy for the identification of T_EM_1, T_EM_2, T_EM_17, and T_EM_17.1 according to the expression of chemokine receptors. The CD4^+^T_EM_ cell subset (CD4^+^CCR7^−^CD45RO^+^) was gated for T_EM_1 cells (CD4^+^CD45RO^+^CCR7^−^CCR6^−^CXCR3^+^CCR4^−^), T_EM_2 cells (CD4^+^CD45RO^+^CCR7^−^CCR6^−^CXCR3^−^CCR4^+^), T_EM_17 cells (CD4^+^CD45RO^+^CCR7^−^CCR6^+^CXCR3^−^CCR4^+^), and T_EM_17.1 cell (CD4^+^CD45RO^+^CCR7^−^CCR6^+^CXCR3^+^CCR4^−^) subsets. **B** The summary data of the percentages of CD4^+^T_EM_ cell subsets in the blood of patients with active MPO-AAV patients and HC (n MPO-AAV=33 HC=20). **C** Serum CCL20 level in active MPO-AAV patients and HC was evaluated by ELISA (n MPO-AAV=33 HC=20)

CD4^+^ T_EM_ cells play important roles in immune responses that produce distinct sets of cytokines, present distinct patterns of homing, and elicit different effects [16, 31]. Disparate chemokine receptor expression patterns could be used to identify major CD4^+^ T_EM_ subsets [19, 32]. The total CD4^+^ T_EM_ cell population was subdivided into the CXCR3^+^CCR4^−^CCR6^−^ (T_EM_1), CXCR3^−^CCR4^+^CCR6^−^ (T_EM_2), CXCR3^−^CCR4^+^CCR6^+^ (T_EM_17), and CXCR3^+^CCR4^-^CCR6^+^ (T_EM_17.1) subsets. In contrast to the patterns in PR3-AAV patients in remission identified by Lintermans [19], a decreasing trend in the percentage of T_EM_17 cells and increases in the percentages of T_EM_1 and T_EM_17.1 cell were detected in MPO-AAV patients in active status compared to HC (Fig. 4). In addition, a significantly increased level of CCL20 was found in patients with active MPO-AAV. Accordingly, our data suggested that T_EM_17 cells are probably recruited to sites of inflammation through the CCL20-CCR6 axis during active MPO-AAV.

### Result 5: Associations of CD4^+^ T cell subpopulations and CD8^+^ T cell subsets with clinical characteristics

**Fig. 5:**
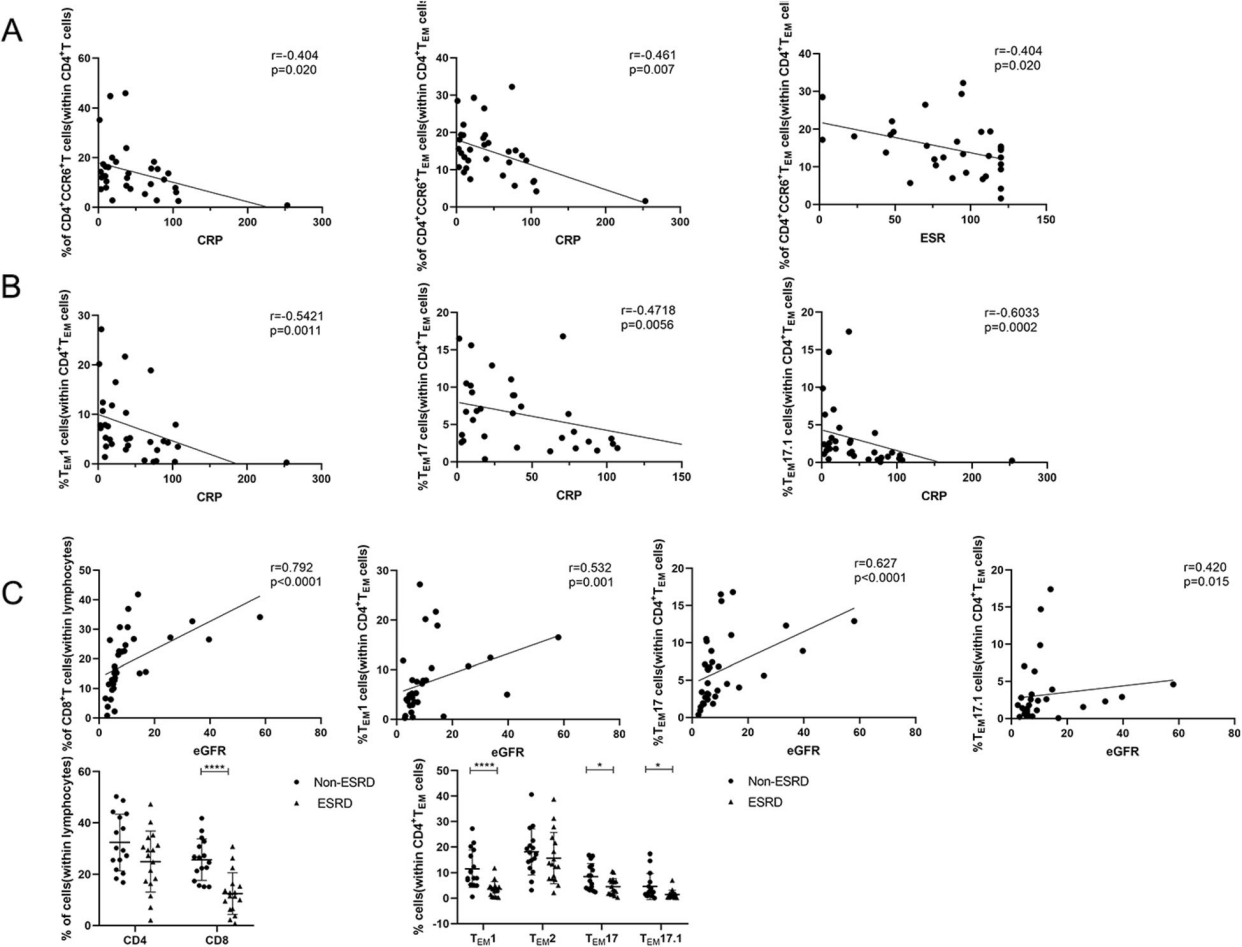
Correlations of the T cell subsets with clinical laboratory parameters in active MPO-AAV patients. **A**, **B** Frequencies of numerous T cell subsets were significantly associated with CRP or ESR using Spearman’s coefficient of correlation, and no obvious correlation was found between T cell subsets and other parameters of disease activity, including platelet count and BVAS (data not shown). **C** The correlations of CD4^+^T cells, CD8^+^T cells, and T_EM_ cell subsets with eGFR and ESRD were shown (n Non-ESRD=16 ESRD=17)

We next evaluated whether changes in the distribution of CD4^+^ T cell subsets and CD8^+^ cell subsets correlated with disease activity and renal outcomes in MPO-AAV patients. The analysis demonstrated that higher CRP levels were associated with lower percentages of CD4^+^CCR6^+^ T cells and CD4^+^CCR6^+^ T_EM_ cells and that a higher ESR was associated with a lower percentage of CD4^+^CCR6^+^ T_EM_ cells (Fig. 5A). The subsets of T_EM_1 and T_EM_17 T_EM_17.1 cell were also negatively correlated with CRP (Fig. 5B). ESRD was defined as dialysis dependence for greater than 3 months, which can be used to indicate renal survival. Here, our results were consistent with those of previous studies [5, 9, 10, 33–36], which have reported that CD8^+^ T cells, Th1 cells, and Th17 cells have pathogenic roles and mediate glomerular injury in AAV. Our results indicated that eGFR has a positive relationship with the proportions of CD8^+^ T, T_EM_1, T_EM_17, and T_EM_17.1 cell. Similarly, reduced peripheral proportions of CD8^+^ T, T_EM_1, T_EM_17, and T_EM_17.1 cell were associated with poor renal outcomes (Fig. 5C).

## Discussion

It is generally accepted that cellular immunity is important and has been shown to be a major component in inducing inflammatory injury in AAV pathogenesis [4]. Abnormities of T cell subsets in the peripheral blood have been reported in PR3-AAV [23, 37]. Although there is considerable overlap, many differences exist between patients with PR3-AAV and MPO-AAV including epidemiology, genetic features, histopathologic features, and clinical features [1]. So far, reports about the distribution of T cells in MPO-AAV are comparatively limited especially in China. In this study, we show for the first time that alterated circulating CD4^+^ T and CD8^+^ T cell subsets and their receptors were associated with disease activity and kidney survival in patients with active MPO-AAV.

Decreases in lymphocytes and CD4^+^ T cells in the peripheral blood have been found in our study, which was consistent with the study in PR3-AAV [23, 37]. Besides, a significant decrease of CD8^+^T cells was first found in our study. Numbers of reason cause peripheral lymphopenia, including infection, neoplasms, standard induction therapy or other autoimmune complications, and intense recruitment of lymphocytes within inflammatory tissue [38]. To avoid interfering by these factors, we established strict exclusion criteria and included newly diagnosed and active MPO-AAV patients, without using immunosuppressive treatment. Therefore, we sepeculated these changes may be attributed to the selective recruitment of T cells into inflamed tissues. In fact, Wacrenier et al. have reported that lymphopenia correlates with the severity of AAV glomerulonephritis at diagnosis and predicts poor renal outcome [39]. O’Sullivan et al. have demonstrated that tubulointerstitial infiltration numbers of CD4^+^T and CD8^+^T cells correlated with lower eGFR [35]. Similar to these studies, we also found a positive correlation between CD8^+^T cells in the peripheral blood and eGFR. Besides, the peripheral proportions of CD4^+^T cells and CD8^+^T cells were lower in active MPO-AAV patients needing renal replacement therapy.

As is known to all, chemokine receptors that are expressed on different T cell subsets play important role in mediating distinct inflammatory migration patterns of T cells [40]. Therefore, we analyzed the expression of correlative chemokine receptors on CD4^+^ and CD8^+^ T cells. We observed decreased CCR6^+^ cell frequencies and increased CXCR3^+^ cell frequencies, but no changes in CCR4^+^ cell frequencies within the total CD4^+^ and CD8^+^T cells in the peripheral blood of patients with active MPO-AAV. Inconsistent with the findings of Fagin et al. [17], their study showed significant increased in the frequencies of circulating CCR4^+^ and CCR6^+^ cell within the total CD4^+^ T cell population in PR3-AAV in active phase and remission. Besides, the increased expression of CCR4 and CCR6 was largely limited to T_EMRA_ and T_CM_ subsets [17]. However, the expression of chemokine receptors on CD8^+^ T cells in AAV has not been investigated. To explain this discrepancy, we propose two hypotheses: (1) MPO-AAV differs from PR3-AAV in the pathological mechanism. (2) All patients we included were in the active phase, while the data of a previous study by Fagin et al. were obtained from a collection of PR3-AAV patients in either the active phase or remission.

Moreover, alterations of CCR6^+^ and CXCR3^+^ cells in our study were mainly confined to T_EM_ and T_EMRA_ cells. Notably, T_EM_ cells are generally considered as the main executor of adaptive immunity and have been reported to play important roles in inflammation and organ damage in AAV [41]. Ruth et al. found that mice depleted of effector CD4^+^ T cells developed attenuated crescent formation and effector cell influx in experimental anti-MPO crescentic glomerulonephritis [9]. Sakatsume et al. found that CD45RA^−^ CD45RO^+^ were the mainly phenotypic features of effector T cells in urine from AAV patients with renal damage. In line with these finding, numerous T cells that infiltrated in active lesion of human ANCA-associated glomerulonephritis were also effector type [42]. In agreement with the previous results [43, 44], we found that the level of CCL20 (the ligand of CCR6) in the peripheral blood was significantly increased in active MPO-AAV patients. Considerable studies have reported the involvement of effector memory T cells in many inflammatory and autoimmune diseases via chemokine-chemokine receptor pathway [18, 28–30]. Given our findings here, the reduction of CCR6^+^T_EM_ cells, CCR6^+^T_EMRA_ cells were very likely associated with the migration toward inflamed tissues though CCL20-CCR6 axis.

In this study, the expansion of T_EM_1 cells in patients with active MPO-AAV reflected a skewed T_EM_1 immune response, which was similar to Lúdvíksson et al. [45] and Lamprecht et al. [46] reports that a predominant Th1 response was reported in patients with localized or active PR3-AAV. In addition, we observed an increased frequency of T_EM_17.1 cell in the peripheral blood of patients with active MPO-AAV compared to HC, which has already been reported in patients with AAV [47, 48]. Inconsistent with previous studies that showed sustained Th17 cell expansion in PR3-AAV patients independent of disease activity [44, 48], we did not find an increased proportion of T_EM_17 cells in patients with active MPO-AAV. Instead, a decreasing trend was observed. Significantly, there is controversy about the peripheral distribution of Th17 cells in AAV. Some reports did not identify a skewed Th17 response [43, 49]. Lilliebladh et al. found no differences in the percentages of Th17 cells in patients with MPO-AAV, similar to our results [50].

Several lines of evidence for the implication of Th1 and Th17 in AAV exist. Abundant IL-17-producing cells were detected in renal biopsies of patients with active necrotizing and crescentic ANCA-associated glomerulonephritis. The major source of IL-17 is neutrophil, and IL-17^+^T cells are only present at lower frequencies. Nevertheless, these IL-17^+^T cells are significantly correlated with serum creatinine level [36]. The renal IFN-γ-producing T cell infiltration has not yet investigated. In murine anti-MPO glomerulonephritis, Th17 and Th1 cells were demonstrated to promote the development of autoimmune renal damage [10, 33]. Consistent with these previous studies, we found that the peripheral proportions of T_EM_1, T_EM_17, and T_EM_17.1 cell positively correlated with the eGFR. Furthermore, these subsets in the ESRD group were significantly lower than those in the non-ESRD group of patients with MPO-AAV. Besides, the frequency of circulating T_EM_1, T_EM_17, and T_EM_17.1 cell were negatively correlated with CRP. These results implied that these aberrant T cell subsets might be potential markers to evaluate disease activity and predict renal survival. Significantly, a study on experimental murine anti-MPO glomerulonephritis model found that the renal involvement of T helper subset is biphasic, the dominance of the Th17 subset during the development of early autoimmunity followed by Th1 dominance in late autoimmunity [10]. Consequently, we speculated that the decrease in T_EM_17 cells accompanied by increases in T_EM_1 and T_EM_17.1 cells in the peripheral blood of patients with active MPO-AAV in our study were due to dynamic development of anti-MPO autoimmunity. However, study on the time kinetics and cellular effectors pattern of renal and systemic Th1 and Th17 immune responses in human anti-MPO autoimmunity is still lacking. Therefore, more studies are needed to observe the characteristic of T helper cell responses in the natural course of ANCA-associated glomerulonephritis.

## Conclusion

Our study showed dysregulated chemokine receptor expression on CD4^+^ and CD8^+^ effector memory T cells and aberrant distribution of functional CD4^+^ T cell subsets in the peripheral blood of active MPO-AAV patients, which correlates with clinical and renal severity. Dysfunction of chemokine receptor expression could predict a poor renal outcome and disease activity in patients with MPO-AAV.

## Data Availability

The datasets used and analyzed during the current study are available from the corresponding author on reasonable request.

## Abbreviations

ANCA: Antineutrophil cytoplasmic antibody
AAV: ANCA-associated vasculitis
MPO: Myeloperoxidase
PR3: Proteinase 3
HC: Healthy control
PBMCs: Peripheral blood mononuclear cells
eGFR: Estimated glomerular filtration rate
T_naïve_: Naïve T cell
T_EM_ cell: Effector memory T cell
T_CM_ cell: Central memory T cell
CCR: CC chemokine receptor
CXCR: CXC chemokine receptor
BAVS: Birmingham vasculitis activity score
ESRD: End-stage renal disease
ELISA: Enzyme-linked immunosorbent assay
CCL: CC chemokine ligand
CRP: C-reactive protein
ESR: Erythrocyte sedimentation rate
Th cells: T helper cells
T_EMRA_: Effector memory T cells re-expressing CD45RA
IFN-γ: Interferon gamma
TNF-α: Tumour necrosis factor alpha
